# Deletion of Tricellulin Causes Progressive Hearing Loss Associated with Degeneration of Cochlear Hair Cells

**DOI:** 10.1038/srep18402

**Published:** 2015-12-18

**Authors:** Toru Kamitani, Hirofumi Sakaguchi, Atsushi Tamura, Takenori Miyashita, Yuji Yamazaki, Reitaro Tokumasu, Ryuhei Inamoto, Ai Matsubara, Nozomu Mori, Yasuo Hisa, Sachiko Tsukita

**Affiliations:** 1 Laboratory of Biological Science, Graduate School of Frontier Biosciences and Graduate School of Medicine, Osaka University, Osaka 565-0781, Japan; 2 Department of Otolaryngology-Head and Neck Surgery, Kyoto Prefectural University of Medicine, Kyoto 602-8566, Japan; 3 Department of Otolaryngology, Faculty of Medicine, Kagawa University, Kagawa 761-0793, Japan; 4 Present address: Osaka Bay Central Hospital, Osaka 552-0021, Japan

## Abstract

Tricellulin (also known as MARVELD2) is considered as a central component of tricellular tight junctions and is distributed among various epithelial tissues. Although mutations in the gene encoding tricellulin are known to cause deafness in humans (DFNB49) and mice, the influence of its systemic deletion *in vivo* remains unknown. When we generated *tricellulin*-knockout mice (*Tric*^−/−^), we found an early-onset rapidly progressive hearing loss associated with the degeneration of hair cells (HCs); however, their body size and overall appearance were normal. *Tric*^−/−^ mice did not show any morphological change pertaining to other organs such as the gastrointestinal tract, liver, kidney, thyroid gland and heart. The endocochlear potential (EP) was normal in *Tric*^−/−^ mice, suggesting that the tight junction barrier is maintained in the stria vascularis, where EP is generated. The degeneration of HCs, which occurred after the maturation of EP, was prevented in the culture medium with an ion concentration similar to that of the perilymph. These data demonstrate the specific requirement of tricellulin for maintaining ion homeostasis around cochlear HCs to ensure their survival. The *Tric*^−/−^ mouse provides a new model for understanding the distinct roles of tricellulin in different epithelial systems as well as in the pathogenesis of DFNB49.

Tight junctions (TJs) form close intercellular connections between epithelial cells and maintain epithelial barrier function by regulating paracellular permeability. On observation using electron microscopy, TJs appear as intramembranous anastomosing chains of particles called TJ strands^1^. There are two subtypes of TJs with complementary function at cellular contacts between either two or three cells. TJs between two adjacent cells, called bicellular TJs (bTJs), form a horizontal band of strands, and TJs at tricellular junctions, called tricellular TJs (tTJs), form vertical strands^1^. Claudins, which belong to the family of tetraspan membrane proteins, and tricellulin (also known as MARVELD2) are the major cell–cell adhesion molecules of the bTJ and tTJ strands, respectively^2^. Although tricellulin localises mainly to tTJs and sparsely to bTJs, it is considered to play a key role in the organisation and function of both tTJs and bTJs. *Tric* knockdown in cell lines causes loosened tTJs, thinner bTJ belts^2^ and an increase in the paracellular ion permeability, which is decreased when tricellulin is overexpressed^3^.

The cochlea is the sensory organ responsible for the hearing that is compartmentalised into two spaces filled with fluids having different ion compositions; perilymph and endolymph. The perilymph contains low and high concentrations of K^+^ (4.2 mM) and Na^+^ (148 mM) ions, respectively, which is common for general extracellular fluids, whereas the endolymph contains extraordinarily high concentrations of K^+^ (157 mM) and low concentrations of Na^+^ (1.3 mM) ions^4^. The endolymph retains endocochlear potential (EP), which provides the driving force for generating K^+^ current to depolarise sensory epithelial cells, called hair cells (HCs), upon acoustic stimulation. EP is generated in the stria vascularis, an isolated compartment facing endolymphatic and perilymphatic spaces^5^,^6^. HCs in the organ of Corti, which is the cochlear sensory epithelium, are divided into inner hair cells (IHCs) and outer hair cells (OHCs). Both OHCs and IHCs share a common signal transduction mechanism that functions at the stereocilia on the apical surface with different roles as follows: IHCs transmit sound-evoked electrical signals, whereas OHCs amplify mechanical signals through its somatic motility. The apical surface of HCs contacts the endolymph and the basolateral membrane is soaked in the perilymph.

TJ is considered crucial for the cochlear function because mutations or lack of TJ-associated proteins, such as claudin 9, 11, 14 and occludin cause deafness in humans and/or mice^7^,^8^,^9^,^10^,^11^,^12^,^13^. In addition, the essential role of tricellulin in the cochlea has been recently noted^14^,^15^,^16^,^17^,^18^. Mutations in *TRIC* cause human non-syndromic hearing loss (DFNB49)^14^,^15^,^16^,^17^ and *Tric*-knockin mice carrying a mutation orthologous to DFNB49 show a similar auditory phenotype^18^. However, it is not clear if the auditory phenotype in DFNB49 and the *Tric*-mutated mice is due to the partial or total loss of the tricellulin function. Moreover, *Tric*-mutated mice show significantly increased body weight and abnormal morphology in various organs^18^, suggesting the additional systemic effect of the mutated tricellulin.

In the current study, we demonstrate the effect of the systemic total deficiency in tricellulin by generating *Tric*-knockout (KO) mice (*Tric*^−/−^ mice). These mice show hearing loss associated with the degeneration of HCs, which resembles the phenotype of DFNB49 and the *Tric*-mutated mice, without apparent morphological change in other organs. These results will provide a new basis for understanding the role and redundancy of the tricellulin function.

## Results

### The generation and comprehensive phenotypic characterisation of *Tric* ^−/−^ mice

**Figure 1:**
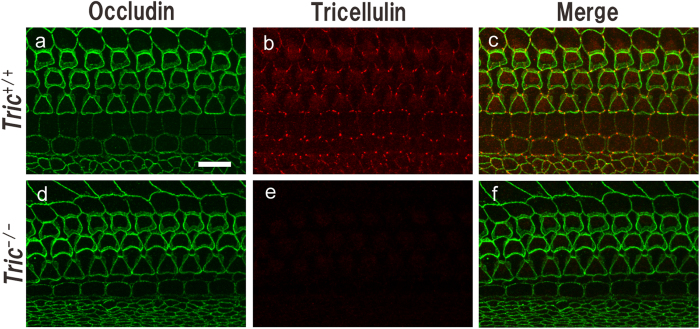
Localisation of tricellulin in the organ of Corti. Organ of Corti harvested from P8-*Tric*^+/+^ (**a**–**c**) and *Tric*^−/−^ mice (**d**–**f**) were immunostained using antibodies against occludin (**a,c,d,f**) and tricellulin (**b,c,e,f**). The overall geometries of apical junctions are comparable between *Tric*^+/+^ (**a**) and *Tric*^−/−^ mice (**c**). Tricellulin is localised mainly to tricellular TJs in *Tric*^+/+^ mice (**b**) but is undetectable in *Tric*^−/−^ mice (**e**). Scale bar = 10 μm.

We generated *Tric*^−/−^ mice through homologous recombination in TT2 ES clones using a targeting vector to replace exon 2 of *Tric* with a cassette encoding neomycin resistance (Fig. S1a). Because the exon 2 encodes all of the 4 transmembrane domains and its deletion leads to a frameshift mutation, functional tricellulin cannot be produced in *Tric*^−/−^ mice. Polymerase chain reaction (PCR) analysis demonstrated the disruption of *Tric* (Fig. S1b), and reverse-transcription PCR (RT-PCR) and western blotting analyses did not detect *Tric* mRNA or tricellulin, respectively, in the colon tissue extracts of *Tric*^−/−^ mice on postnatal day (P) 30-P60 (Fig. S1c,d). The immunoreactivity of tricellulin of P8-*Tric*^−/−^ mice was undetectable in the organ of Corti (Fig. 1), stria vascularis, Reissner’s membrane and utricle (Fig. S2) without gross morphological alterations of tTJs and bTJs demonstrated by immunohistochemical analyses of occludin or ZO-1 expression.

*Tric*^−/−^ mice were fertile, and their offspring were born in the expected Mendelian ratios. We did not detect changes in the general appearance of *Tric*^−/−^ mice or in the levels of biochemical serum markers (Table S1). The body weight of P45 *Tric*^−/−^ mice (17.9 ± 0.6 g, n = 4) was comparable to that of *Tric*^+/+^ littermates (16.9 ± 0.7 g, n = 4, p = 0.28, two-tailed unpaired Student’s *t* test). The histology of tissues, including the colon, small intestine, liver, kidney, thyroid gland and heart, is not distinguishable between *Tric*^−/−^ and *Tric*^+/+^ mice (Fig. S3).

### *Tric* ^−/−^ mice suffer an early-onset rapidly progressive hearing loss

**Figure 2:**
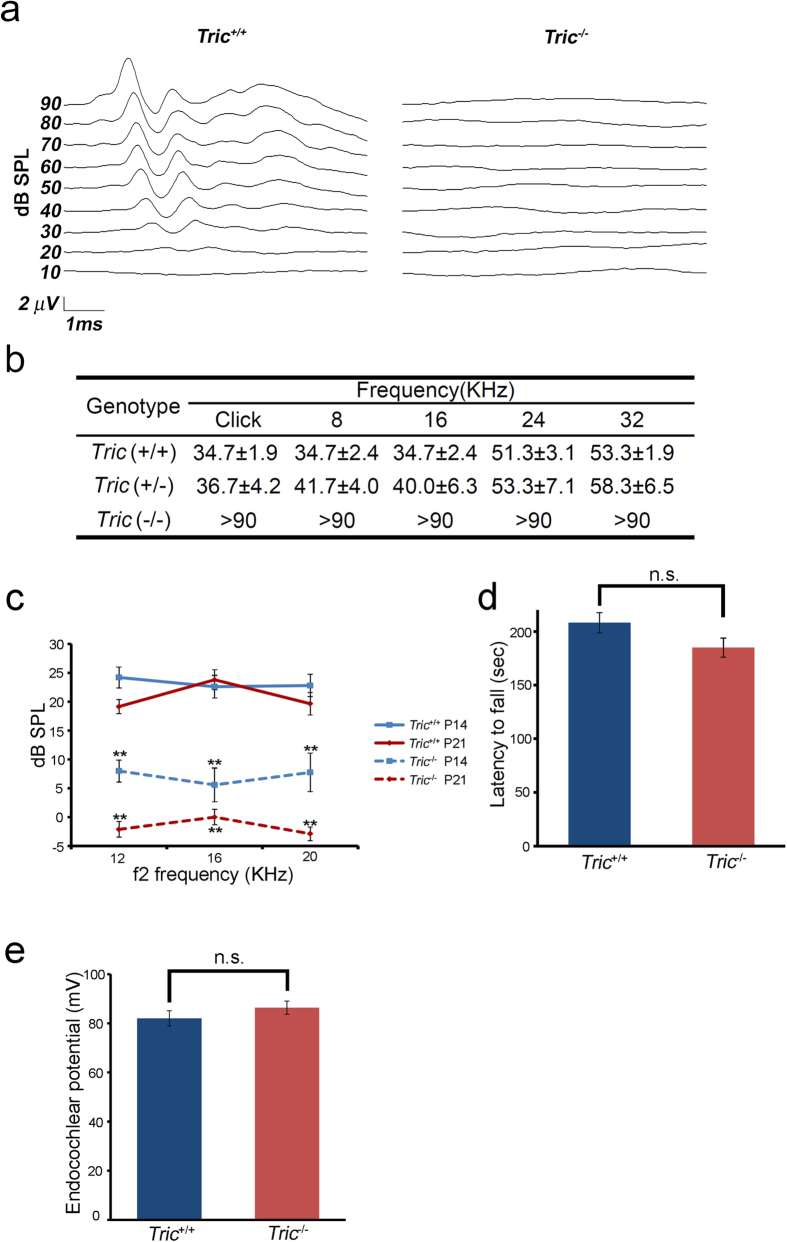
Progressive hearing loss and normal EP of *Tric*^−/−^ mice. (**a**) Representative ABR waveforms to click stimuli for P21-*Tric*^+/+^ and *Tric*^−/−^ mice. (**b**) ABR thresholds of *Tric*^+/+^, *Tric*^+/−^ and *Tric*^−/−^ mice in response to a broadband click and to tone-burst stimuli of 8, 16, 24 or 32 kHz; n = 15 (*Tric*^+/+^), 6 (*Tric*^+/−^) and 12 (*Tric*^−/−^). *Tric*^−/−^ mouse did not generate detectable waves in response to any stimulus, while *Tric*^+/+^ and *Tric*^+/−^ mice did not show a significant difference; P = 0.639 (click), 0.135 (8 kHz), 0.468 (16 kHz), 0.797 (24 kHz) and 0.648 (32 kHz). (**c**) DPOAE for 12, 16 and 20 kHz on P14 (blue) and P21 (red) of *Tric*^+/+^ (solid lines) and *Tric*^−/−^ (dotted lines) mice. Note the reduced response on P14 and no response on P21 of *Tric*^−/−^ mice; n = 10 (P14) and 16 (P21), P < 0.01 at all frequencies. (**d**) Rotarod test of *Tric*^+/+^ and *Tric*^−/−^ mice on P60-P90 mice. *Tric*^−/−^ mice showed normal motor and balance function. (**e**) EP of *Tric*^+/+^ and *Tric*^−/−^ mice on P17–24.

When we analysed hearing by observing the startle response elicited by a sound, i.e. Preyer’s reflex, we found that *Tric*^−/−^ mice on P21–P60 lacked the reflex, although *Tric*^+/+^ and *Tric*^+/−^ littermates responded. We then measured hearing using the auditory brainstem response (ABR), a sound-induced potential in the auditory pathway from the cochlea to brainstem, on P14 when the hearing function is initially detectable in wild-type mice. P14-*Tric*^−/−^ mice exhibited severe hearing loss, with a click ABR threshold of 87.5 ± 3.2 dB sound pressure level (SPL; n = 16), which was significantly higher compared with that in *Tric*^+/+^ mice (60 ± 4.2 dB SPL, n = 8, P = 0.00009. data not presented as figures). P21-*Tric*^−/−^ mice suffered from profound hearing loss with no detectable ABR using a 90 dB click or tone burst stimuli at 8, 16, 24 or 32 kHz (Fig. 2a,b). In contrast, *Tric*^+/−^ mice maintained a response comparable with that of *Tric*^+/+^ mice. We evaluated the distortion product otoacoustic emission (DPOAE), which represents low-level sound generated by the somatic motility of OHCs and emitted to the external auditory canal, of mice on P14 and P21 (Fig. 2c). DPOAE of *Tric*^−/−^ mice was significantly smaller compared with that of *Tric*^+/+^ mice at all frequencies measured on P14 and was undetectable on P21. Despite the apparent hearing disability, *Tric*^−/−^ mice had no detectable impairment of balance and exhibited normal gait. Rotarod test showed the normal motor and balance function of *Tric*^−/−^ mice (Fig. 2d). The latency to fall of *Tric*^+/+^ and *Tric*^−/−^ mice were 208 ± 9.3 s and 184.9 ± 8.9 s, respectively (n = 5, p = 0.11).

### EP and the paracellular permeability of the stria vascularis are maintained in *Tric* ^−/−^ mice

The hearing loss observed in *Tric*^−/−^ mice was likely due to decreased EP because the TJ in the stria vascularis contains tricellulin (Fig. S2a–f), and the dysfunction of TJs in the stria vascularis reduces EP^9^,^10^. To test this possibility, we measured EP of *Tric*^−/−^ mice on P17–P24 and compared with that of *Tric*^+/+^ littermates. Unexpectedly, there was no significant difference in EP between *Tric*^+/+^ (82.0 ± 3.1 mV, n = 11) and *Tric*^−/−^ mice (86.4 ± 2.7 mV; n = 8; P = 0.325; Fig. 2e). Using biotin (molecular weight = 556.59) as a tracer, we tested whether permeability from the perilymph to the stria vascularis increased in *Tric*^−/−^ mice because TJs in the stria vascularis are impermeable to biotin in wild-type mice but are permeable in *claudin 11*-KO mice, in which bTJs in the stria vascularis are disrupted^9^. Biotin perfused into the perilymph of *Tric*^−/−^ and *Tric*^+/+^ mice was detected in the intact spiral ligament, which is in contact with the perilymph and located adjacent to the stria vascularis. In contrast, biotin was excluded from the stria vascularis of *Tric*^−/−^ and *Tric*^+/+^ mice (Fig. S4a,b).

### Apoptosis causes the progressive degeneration of cochlear HC in *Tric* ^−/−^ mice

We next examined the morphology of the cochlea because disruption of specific TJ proteins affects the survival of HCs^7^,^8^,^11^. In haematoxylin–eosin (HE) stained *Tric*^−/−^ cochlea on P21, HCs were frequently lost without morphological change in other regions of the cochlea, such as the spiral ganglion and stria vascularis (Fig. S5a–d). Therefore, we focused on the morphology and survival of cochlear HCs in *Tric*^−/−^ mice.

**Figure 3:**
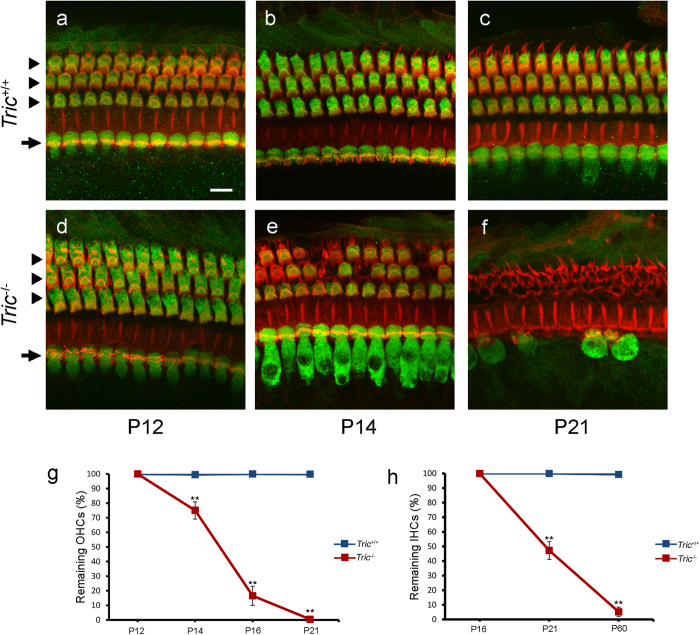
Progressive degeneration of HCs in the organ of Corti of *Tric*^−/−^ mice. (**a**–**f**) Middle turn of the organ of Corti of *Tric*^+/+^ (**a**–**c**) and *Tric*^−/−^ (**d**–**f**) mice on P12 (**a**,**d**), P14 (**b**,**e**) and P21 (**c**,**f**) stained with a HC marker myosin VIIa (green) and rhodamin phalloidin (red). IHCs (arrows) and OHCs (arrowheads) are regularly aligned in *Tric*^+/+^ cochlea until P21 (**a–c**). Hair cells in *Tric*^−/−^ cochlea are normal on P12 (**d**). OHCs progressively degenerate at P14 (**e**) to P21 (**f**), and some IHCs degenerate at P21 (f). Scale bar = 10 μm. Progressive loss of OHCs (**g**) and IHCs (**h**) from the cochlea of *Tric*^−/−^ mice. The loss of HCs is not detected in *Tric*^+/+^ cochlea (blue lines). The degeneration of HCs of *Tric*^−/−^ mice (red lines) progresses during P12–P21 in OHCs (**g**) and P16–P60 in IHCs (**h**).

We analysed the organ of Corti dissected from the middle turn of the cochlea of P12, P14, P16, P21 and P60 mice using phalloidin staining together with labelling of the HC-specific marker myosin VIIa. In *Tric*^+/+^ mice, a single row of IHCs and three rows of OHCs were regularly aligned, and the loss of HCs was not observed (Fig. 3a–c). The morphology of HCs of P12-*Tric*^−/−^ mice was indistinguishable from that of *Tric*^+/+^ littermates (Fig. 3d). However, OHCs were occasionally lost by P14 (Fig. 3e), which rapidly progressed with age, and were completely lost by P21 (Fig. 3f,g). The ratio of remaining OHCs in *Tric*^+/+^ and *Tric*^−/−^ mice was 99.6 ± 0.4 *vs* 75.0 ± 5.9% on P14, 100.0 ± 0.0 *vs* 16.5 ± 6.5% on P16 and 99.8 ± 0.2 *vs* 0.5 ± 0.5% on P21 (n ≥ 5, P = 0.0008 on P14; n ≥ 4, P < 0.001 on P16; n = 5, P < 0.001 on P21; Fig. 3g). The loss of IHCs was first detected on P21 (Fig. 3f), and most IHCs were lost on P60. The ratio of remaining IHCs in *Tric*^+/+^ and *Tric*^−/−^ mice were 100.0 ± 0.0 vs 47.3 ± 6.1% on P21 and 99.4 ± 0.6 vs 5.2 ± 3.2% on P60 (n ≥ 6, P < 0.001 on P21 and n ≥ 3, P < 0.001 on P60; Fig. 3h). In contrast, the degeneration of HCs of *Tric*^−/−^ mice was not detected in vestibular sensory organs, such as the crista ampullaris or utricular macula (Fig. S6).

**Figure 4:**
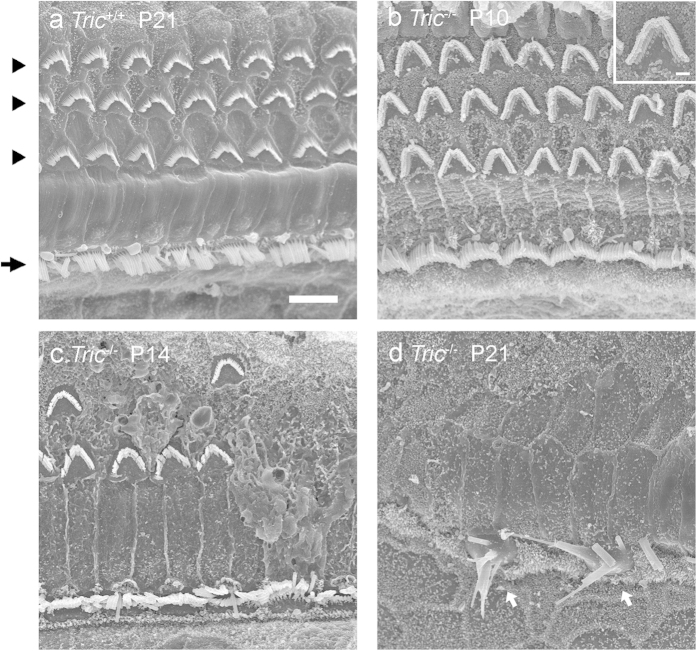
Ultrastructure of degenerated HCs of *Tric*^−/−^ mice. (**a**–**d**) SEM images of the organ of Corti of *Tric*^+/+^ (**a**) and *Tric*^−/−^ (**b**–**d**) mice. (**a**) IHCs (arrow) and OHCs (arrowheads) with normally organised arrays of stereocilia are observed in *Tric*^+/+^ mice on P21. (**b**) Morphology of HCs is normal in *Tric*^−/−^ cochlea on P10. Note the V-shaped array of stereocilia in OHCs (inset). (**c**) Degeneration occurs frequently in OHCs but not in IHCs of *Tric*^−/−^ cochlea on P14. (**d**) Most HCs are lost except for some IHCs with severely deformed stereocilia (arrows) in *Tric*^−/−^ cochlea on P21. Scale bars = 6 μm (**a–d**) and 1 μm (inset in a).

Scanning electron microscopy (SEM) was used in an attempt to detect ultrastructural changes in HCs and the organ of Corti on P10, P14 and P21. In *Tric*^+/+^ mice, the alignment of IHCs and OHCs and the characteristic array of stereocilia, which is slightly curved or linear in IHCs and V shaped in OHCs, were maintained normally until P21 (Fig. 4a). As we expected, HCs of *Tric*^−/−^ mice were normal on P10 (Fig. 4b). Some OHCs disappeared on P14, although the normal array of stereocilia was usually maintained in remaining OHCs (Fig. 4c). On P21, all OHCs and most IHCs were lost and replaced by supporting cells as generally observed in the damaged organ of Corti^19^,^20^,^21^, and the stereocilia in remaining IHCs were often fused to form rod-like structures (Fig. 4d).

**Figure 5:**
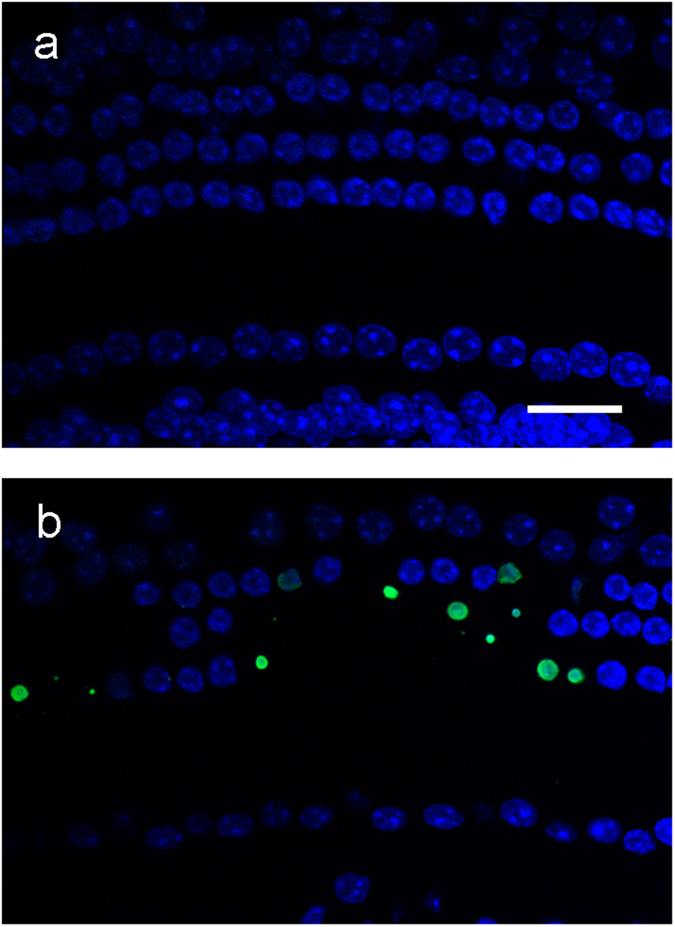
Apoptotic hair cell death of *Tric*^−/−^ cochlea. TUNEL assay of Tric+/+. (**a**) and *Tric*^−/−^ (**b**) cochleae on P15. Nuclei were counterstained with Hoechst 33258 (blue). Note the TUNEL-positive (green) condensed nuclei in *Tric*^−/−^ cochlea. Scale bar = 20 μm.

To determine whether apoptosis contributed to the degeneration of HCs, we performed terminal deoxynucleotidyl transferase-mediated dUTP nick-end labelling (TUNEL) assays of organ of Corti on P15, when loss of OHCs was expected to progress most robustly. TUNEL-positive cells with condensed nuclei were frequently observed in OHCs of *Tric*^−/−^ mice, suggesting that the death of HCs was caused by apoptosis (Fig. 5).

### The ultrastructure of bTJs is maintained in *Tric* ^−/−^ mice

**Figure 6:**
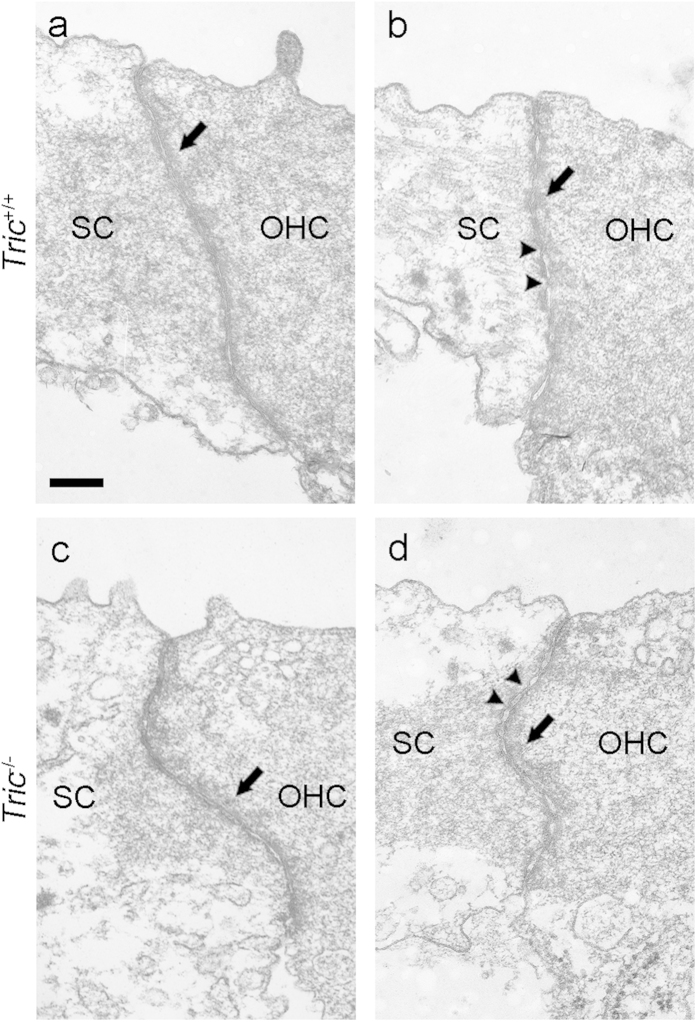
Normal bTJs in *Tric*^*−/−*^ mice. TEM images of bTJs of *Tric*^+/+^ (**a**,**b**) and *Tric*^−/−^ (**c**,**d**) mice. Note the normally-formed membrane contact sites (arrowheads) and perijunctional densities (arrows) in *Tric*^−/−^ mice. SC: supporting cell. Scale bar = 200 nm.

Because tricellulin localises and functions at bTJs as well as at tTJs^2^,^3^, structural disruption of bTJs may impair the tissue integrity in the organ of Corti leading to the observed loss of HCs. Using transmission electron microscopy (TEM), we observed bTJs between an OHC and a neighbouring supporting cell in the cochlea of *Tric*^−/−^ mice on P12–P14. bTJs in *Tric*^−/−^ mice exhibited normally-formed membrane contact sites and perijunctional density, which were indistinguishable from those of *Tric*^+/+^ mice (Fig. 6). However, freeze-fracture electron microscopy did not detect tTJ strands in *Tric*^+/+^ or *Tric*^−/−^ mice, which we attribute to technical limitations.

### Apicobasal polarity is maintained in *Tric* ^−/−^ HCs

TJ establishes cellular apicobasal polarity; therefore, the deficiency of tricellulin may compromise apicobasal polarity in HCs leading to their loss. We used protein kinase C zeta (PKCζ) as a marker of apicobasal polarity in the organ of Corti of *Tric*^−/−^ mice. PKCζ localises to the apical but not to the basolateral surface of the membrane of epithelial cells, including HCs, and is required to establish epithelial polarity and formation of TJs. We found that PKCζ was restricted to the apical surface of HCs and supporting cells of *Tric*^−/−^ mice, which was comparable with those of *Tric*^+/+^ mice, suggesting normal apicobasal polarity of *Tric*^−/−^ HCs (Fig. S7).

### Degeneration of HCs in the cochleae of *Tric* ^−/−^ mice is prevented in explant culture

**Figure 7:**
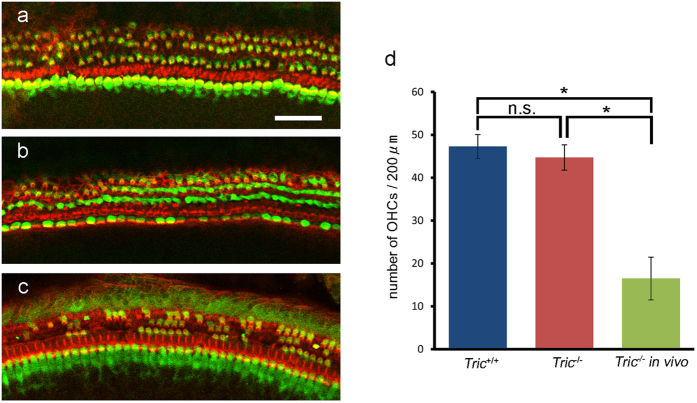
*Tric*^−/−^ HCs survive in explant culture. *Tric*^+/+^ (**a**) and *Tric*^−/−^ (**b**,**c**) cochleae in explant culture (**a**,**b**) and *in vivo* (**c**) stained using an antibody against myosin VIIa (green) and rhodamine phalloidin (red). (**a,b**) Most OHCs survive in *Tric*^+/+^ and *Tric*^−/−^ cochleae cultured until P16. (**c**) Most OHCs are lost on P16 *Tric*^−/−^ cochlea *in vivo*. (**d**) The numbers of remaining OHCs in culture does not differ between *Tric*^+/+^ and *Tric*^−/−^ cochleae, and they are significantly larger compared with those *in vivo*. Scale bars: 50 μm.

To test whether the degeneration of HCs was caused by leakage of highly concentrated K^+^ from the endolymph to perilymph around the basolateral membrane of HCs, we dissected the organ of Corti from the middle turn of the cochlea and cultured explants in medium with an ion composition similar to that of the perilymph. The organ of Corti was harvested on P4 and cultured until P16, which was the stage when abundant degeneration of OHCs was observed *in vivo*. In contrast to *in vivo* observations (16.5 ± 5.0 cells/200 μm, n = 7), significantly larger numbers of OHCs in the explant culture from *Tric*^−/−^ mice survived until P16 (47.3 ± 2.8 cells/200 μm, n = 9, P < 0.001). Moreover, there was no significant difference in the numbers of remaining OHCs between *Tric*^+/+^ and *Tric*^−/−^ mice (44.7 ± 3.0 cells/200 μm, n = 7; Fig. 7).

## Discussion

In the present study, we demonstrated for the first time the effect of systemic deletion of the gene encoding tricellulin from mice and showed that tricellulin deficiency causes an early-onset progressive hearing loss without apparent morphological change in other organs, which resembles the phenotype reported in human DFNB49^14^,^15^,^16^,^17^. We were surprised to find that *Tric*^−/−^ mice did not show apparent histological or functional disturbance in multiple organs except for hearing loss because tricellulin is ubiquitously expressed in various epithelial tissues, such as the intestine, stomach, colon, kidney, epidermis, liver, thyroid and pancreatic duct as well as in endothelial cells^2^,^22^,^23^,^24^,^25^,^26^. In contrast, the body weights of homozygous and heterozygous mice carrying a mutation orthologous to DFNB49 are higher compared with those of wild-type mice, and the tissues of homozygous mice, such as the salivary gland, thyroid gland and heart, are histologically abnormal^18^. Because we failed to detect these phenotypes in *Tric*^−/−^ mice, the prematurely terminated tricellulin protein predicted to be expressed in the *Tric-*mutant mice^18^ may have a pathogenic function. With regard to our screening, the apparent pathological phenotype of *Tric*^−/−^ mice was limited to the cochlea, suggesting that tricellulin has the most indispensable role in cochlea. However, we cannot exclude the possible compensation of tricellulin by unidentified molecules in other tissues.

The cochlea of *Tric*^−/−^ mice developed normally, although they progress into functional and histological degeneration later. ABR and DPOAE measurements indicate that *Tric*^−/−^ mice partially retained hearing on P14, which was rapidly lost by P21. The progressive loss of HCs was the only morphological change observed in the *Tric*^−/−^ cochlea and coincided with the deterioration of hearing during P14–P21, suggesting that the hearing loss was caused by damage to HCs. The loss of HCs in *Tric*^−/−^ mice began much earlier in OHCs than that in IHCs, suggesting that tricellulin was preferentially required by TJs surrounding OHCs. Moreover, deletion of another tTJ protein, immunoglobulin-like domain-containing receptor 1 (ILDR1), causes progressive hearing loss and OHC-dominant HC loss similar to that in *Tric*^−/−^ mice^27^, which further supports the essential role of tTJs in the survival of OHCs. However, *Tric*^−/−^ mice exhibited severe hearing loss on P14 when most HCs were present, suggesting that the function of HCs was disturbed before they died.

*Claudin 11-*KO mice^9^,^10^, in which TJs are specifically disrupted in basal cells of the stria vascularis, show reduced EP and increased biotin permeability in the stria vascularis. In contrast, EP and impermeability to biotin from the perilymph to the stria vascularis were maintained in *Tric*^−/−^ mice, suggesting that tricellulin is not required for the function of epithelial TJs in the stria vascularis. The TJ-based barrier between blood and intrastrial fluid is known to be crucial for maintaining EP^28^. The normal EP of *Tric*^−/−^ cochlea suggests that tricellulin is not required for endothelial TJ integrity of the capillary vessels in the stria vascularis.

The specific requirement for tricellulin in the organ of Corti, particularly at the site where OHCs are located, is likely explained by the continuous exposure of OHCs to intense oscillation generated by the sound as well as its own somatic motility^29^. The oscillation-induced shear force applied to TJs surrounding OHCs may facilitate the disruption of tricellulin-dependent barrier function. Alternatively, tricellulin may exert an unconventional function in the unique heterocellular junction between OHCs and supporting cells, known as the tight-adherens junction that possesses the molecular and structural features of TJs and adherens junctions^30^. For instance, in the cochlea of *Ildr1*-KO mice, tricellulin is mislocalised only in the organ of Corti^31^, suggesting disparate regulation and function of tricellulin among different TJs. However, we cannot exclude the possibility that the OHC-dominant HC death is simply due to the high vulnerability of OHCs to the barrier dysfunction.

An open question is how tricellulin deficiency causes the death of HCs. The gross tissue integrity of the organ of Corti of *Tric*^−/−^ mice was maintained as shown by light microscopy. Immunohistochemical analysis of occludin expression and SEM observations did not show disorganised or separated apical junctions, and TEM observations revealed normally appearing ultrastructures of bTJs in the organ of Corti. The apical localisation of PKCζ in *Tric*^−/−^ HCs suggests the proper maintenance of HC apicobasal polarity. Disruption of tTJ strands may occur in *Tric*^−/−^ mice as it does in *Tric*-mutant mice^18^. Paracellular ion permeability is increased in *Tric*-knockdown Eph4 cells^2^ and decreased in MDCK II cells that overexpress tricellulin^3^. Therefore, increased paracellular ion permeability in the organ of Corti may cause K^+^ to leak from the endolymph to the perilymph around the basolateral membrane of HCs in *Tric*^−/−^ mice. This minor K^+^ leakage may increase the K^+^ concentration in the small perilymphatic space surrounding HCs without affecting EP. Long-term exposure of the basolateral membranes of OHCs to high concentrations of K^+^ causes prolonged depolarisation of OHCs, ultimately leading to cell death^32^. Moreover, degeneration of HCs occurs in mice deficient in KCC3 and KCC4, which are K–Cl co-transporters expressed by supporting cells and required for removing extracellular K^+^ around OHCs^33^,^34^. In an explant culture with ion composition similar to that of the perilymph, HCs of *Tric*^−/−^ mice survived until P16 when most HCs degenerated *in vivo*, supporting the conclusion that altered ion homeostasis led to the death of HCs. Moreover, the degeneration of HCs in *Tric*^−/−^ mice immediately occurs after EP maturation (P8–14)^35^,^36^, suggesting that EP may facilitate K^+^ leakage from the endolymph to the perilymph to initiate the death of HCs. Consistent with this notion, degeneration of HCs was not observed in vestibular system which lacks EP. This hypothesis is further supported by a report that loss of HCs was rescued by deletion of *Pou3f4,* which encodes a transcription factor required for generating EP^37^,^38^ in *Tric*-mutant mice^18^.

Finally, the progressive OHC-dominant loss of HCs in *Tric*^−/−^ mice is similar to the phenotypes reported of mouse lines with gene knockouts or those that express mutant TJ proteins, such as the *claudin 14*-KO^8^, *claudin 9*-mutant^7^, *Tric-*mutant^18^ and *occuldin*-KO^11^, suggesting that the mechanism of HC death is shared by mice with disrupted TJs. Similar to *Tric*^−/−^ mice, normal EP is maintained in *claudin 14*-KO^8^, *claudin 9*-mutant^7^ and *Tric-*mutant^18^ mice, and HC death occurs via apoptosis in *occludin*-KO mice^11^. The initial degeneration of HCs begins during P10–P13 in *claudin 14*-KO mice^8^, on P14 in *claudin 9*-mutant mice^7^, on P16 in *Tric*-mutant mice^18^, on P14 in *Tric*^−/−^ mice and on P12 in *occludin*-KO mice^11^. These differences may reflect varying extents of remaining barrier function.

In summary, we generated *Tric*^−/−^ mice that suffer an early-onset rapidly progressive hearing loss associated with the degeneration of cochlear HCs likely due to the disruption of the epithelial barrier function in the organ of Corti. Because hearing loss was the only pathological phenotype detected in our study and the loss of HCs was the unique morphological change observed in *Tric*^−/−^ mice, we conclude that tricellulin contributes to the integrity of the TJ, most strikingly in the organ of Corti. Further studies are required to identify the distinct roles of tricellulin in different TJs and the mechanism of HC death caused by dysfunction of tricellulin.

## Methods

### Generation of *Tric* ^−/−^ mice

*Tric*^−/−^ mice (Acc. No. CDB0806K http://www2.clst.riken.jp/arg/mutant%20mice%20list.html) were generated as follows: the targeting vector was constructed using a DT-A/*lox*P/PGK-Neo-pA/*lox*P cassette (http://www.cdb.riken.jp/arg/cassette.html) to delete *Tric* exon 2 (Fig. S1a). A diphtheria-toxin expression cassette (Mc1/DT-A) was linked to the 5′ end of the construct for negative selection. TT2 embryonic stem cells were transfected with the targeting vector^39^, and G418-resistant clones were screened for homologous recombination using PCR (forward primer, 5′-GTACTCGGATGGAAGCCGGTCTTGTC-3′, reverse primer 5′-TACTGGCTCTGTGTTAGCCAGGAGC-3′) and Southern blot analysis using 5′ and 3′ probes. Embryonic stem cell clones carrying the targeted *Tric* allele were introduced into ICR 8-cell stage embryos to produce chimeric mice. Chimeric mice with a large embryonic stem cell contribution were crossed with C57BL/6 mice to produce *Tric*^+/−^ mice. *Tric*^+/−^ mice were backcrossed five times to C57BL/6 mice. *Tric*^−/−^ mice were generated by cross-breeding *Tric*^+/−^ mice. They were genotyped using PCR with a mixture of three primers (common forward primer, 5′-GGCCTTCTACTTGCTGCCTGTTAAG-3′, NEO reverse, 5′-GACGTGCTACTTCCATTTGTCACG-3′, and WT reverse, 5′-CTGGGGCCGAATGGTGGCTGTAATG-3′). Amplicons of 814 bp and 554 bp were detected using template DNAs from *Tric*^+/+^ and *Tric*^−/−^ mice, respectively, and were detected in *Tric*^+/−^ mice (Fig. S1b). All animal experiments were performed in accordance with the declaration of Helsinki and protocols approved by the Osaka University School of Medicine Animal Studies and Committee as well as the Animal Committee of the Faculty of Medicine at Kagawa University.

### Western blotting

Colon from P60 *Tric*^+/+^ and *Tric*^−/−^ mice were fixed with 10% trichloroacetic acid (TCA). Samples were frozen and smashed. The samples were separated by SDS-PAGE and transferred onto a polyvinylidene fluoride membrane. After blocking with 5% skim milk in Tris-buffered saline (TBS) for 1 h, the membrane was incubated with the first antibody (monoclonal rat anti-tricellulin, obtained as previously reported^2^), recognising N-terminal residues 24–69 for 1 h at room temperature. After washing with TBS, the membrane was incubated with secondary antibody (ECL Horseradish Peroxidase linked anti-rat or rabbit IgG antibody; GE healthcare, UK) for 30 min at room temperature. After washing, the membrane was developed using the Immobilon Western chemiluminescent HRP substrate (Millipore). The signals were visualised with ImageQuant LAS 4000 mini (GE healthcare). The membrane was then stripped and reblotted with monoclonal mouse anti-β-actin antibody (SIGMA, A5441) to demonstrate equal loading of samples.

### Immunofluorescence microscopy

Cochleae were dissected, and the perilymphatic space was gently perfused with 10% TCA or 4% paraformaldehyde (PFA) fixed using the same fixative for 20 min (TCA) or overnight (PFA) at 4 °C. The organ of Corti (OC), stria vascularis and vestibular tissues were dissected and permeabilised by incubation in 0.2% triton X-100 in phosphate-buffered saline (PBS) for 15 min, blocked with 4% bovine serum albumin (BSA) for 1 h and incubated with primary antibodies as follows: polyclonal rabbit anti-myosinVIIa (Proteus); monoclonal rat anti-occludin (Moc37), monoclonal rat anti-ZO-1, polyclonal rabbit anti-tricellulin (N450), polyclonal rabbit anti-PKCζ (C-20, Santa Cruz Biotechnology, Inc.) for 1–2 h at room temperature. Antibodies against occludin, ZO-1 and tricellulin were generated as previously reported^2^,^40^,^41^. After washing with PBS, samples were incubated with secondary antibodies as follows: Alexa Fluor 488-conjugated donkey anti-rabbit IgG (Jackson ImmunoResearch), Cy3-conjugated donkey anti-rat IgG or Alexa Flour 488-conjugated donkey anti-rat IgG, together with rhodamine phalloidin for 30 min. Samples were mounted on glass slides using DAKO fluorescent mounting medium and observed using an LSM-510 confocal microscope (Carl Zeiss Inc.). Stacked images and orthographic projection images were acquired using Zeiss LSM Image Browser Ver. 4.2.0.121. All images were processed using Adobe Photoshop 7.0.

### TUNEL assay

Apoptosis was detected using the TUNEL method with an Apoptag Fluorescein *In Situ* Apoptosis Detection Kit (S7110, Chemicon). After fixation using 10% TCA, samples were permeabilised in 0.5% Triton X-100 in PBS for 30 min, processed using the supplier’s TUNEL method and then incubated with Hoechst 33258 for 20 min.

### HE staining

The colon, small intestine, liver, kidney, thyroid gland and heart were obtained from *Tric*^+/+^ and *Tric*^*−/−*^ mice on P30 and fixed in 4% PFA. Temporal bones harvested at P21 were decalcified after fixation using 1.25 M ethylenediaminetetraacetic acid (EDTA) in PBS for 2 days at 4 °C. Paraffin-embedded sections were prepared and stained with HE.

### ABR and DPOAE measurements

Mice were anesthetised using pentobarbital. ABR was measured using a TDT System 3 Real-time Signal Processing System and BioSigRP software (Tucker–Davis Technologies, FL, USA). Responses to clicks and to 8, 16, 24 and 32 kHz bursts were recorded for 10 ms using 50–5000 Hz band-pass filter settings, and ABR waveforms from 500 stimuli were averaged. Thresholds were determined in 10 dB SPL steps of decreasing stimulus intensity until distinct ABR wave patterns were not recognised. The maximum sound intensity tested for each frequency was 90 dB SPL. When no ABR response was detected, 100 dB SPL was applied for statistical analysis. DPOAE was measured using CUBeDIS II v2.40 Auditory Diagnostic System combined with HearID software (Mimosa Acoustics, IL, USA). DPOAEs at frequencies of 2f1–f2 were elicited using two primary pure-tone stimuli (f2/f1 = 1.2, f1 and f2 intensities were 65 dB and 55 dB, respectively). A custom plastic ear tip attached to an ER-10 C probe (Etymotic Research, IL, USA) was inserted into the ear canal, and the DPOAE amplitude was measured at f2 frequencies of 12, 16 and 20 kHz and plotted after subtracting baseline noise.

### Rotarod test

A single track rotarod (MK-630B; Muromachi Kikai, Tokyo, Japan) was used to test for balance and motor impairment of P60-P90 *Tric*^+/+^ and *Tric*^−/−^ mice. Before the experiment, the mice were placed onto the rod (30 mm diameter) for 300 s at a constant speed of 10 rpm to familiarise them with the procedure. For testing, the mice were placed onto the rod with constant acceleration from 4 rpm to 40 rpm in 300 s and the time until fall was recorded. Three trials with a 45–min interval were performed.

### Electron microscopy

For SEM, temporal bones were fixed with 2% PFA and 2.5% glutaraldehyde in phosphate buffer. Cochleae were dissected and fixed with 1% osmium tetroxide (OsO_4_) for 1 h on ice. Samples were incubated in 1% tannic acid for 15 min three times, incubated in OsO_4_ for 40 min, dehydrated and then lyophilised (ES-2030, Hitachi, Japan). After removing the tectorial membrane, samples were coated with OsO_4_ (Neoc-cs, Meiwa, Japan) and examined using an SEM (Hitachi S-4800). For TEM, temporal bones were fixed with 2% PFA and 2.5% glutaraldehyde dissolved in 4-(2-hydroxyethyl)-1-piperazineethanesulphonic acid (HEPES) buffer. After dissection, samples were fixed with 1% OsO4 and then immersed in 0.5% uranyl acetate at room temperature for 2 h. After dehydration, samples were embedded in epoxy resin and ultrathin sections were prepared. The sections were counterstained with uranyl acetate and lead citrate. Specimens were examined using a TEM (JEOL JEM-1010).

### Cultures of the organ of Corti

Mice were decapitated on P4, and the organs of Corti were dissected in Leibovitz’s L-15 medium. The tissues were adhered to petri dishes coated with BD CellTak (BD Life Sciences) and filled with Dulbecco’s modified eagle’s medium (DMEM)/F12 (1:1), which contains 136.34 mM Na^+^ and 4.16 mM K^+^ supplemented with 7% foetal bovine serum. Cultures were maintained at 37 °C in an atmosphere containing 5% CO_2_ for 12 days and then fixed with 4% PFA and processed for immunohistochemical analysis of myosin VIIa using rhodamine phalloidin.

### Tracer permeability assay

Temporal bones were removed from P12 mice. The round and oval windows were opened in PBS containing 1 mM CaCl_2_, and perilymphatic spaces were perfused with 100 ml of EZ-link Sulfo-NHS-LC-Biotin (molecular weight 556.59, 10 mg/ml; Pierce) in PBS for 5 min^9^. After washing with PBS containing CaCl_2_, cochleae were fixed with 10% TCA for 1 h at 4 °C and decalcified with 1.25 M EDTA in PBS for 2 days at 4 °C. Samples were substituted with a graded sucrose series and then incubated with a mixture of 1:1 Tissue-Tek optimal cutting temperature (OCT) compound (Sakura Finetek Japan CO., Ltd., Tokyo) and 20% sucrose overnight at 4 °C. After embedding in the OCT compound, 12-μm thick frozen sections were prepared. After permeabilisation and blocking, biotin was detected using a streptavidin–Alexa Fluor 488 conjugate. 4’,6-diamidino-2-phenylindole was used to stain nuclei.

### Measurement of EP

Animals were anesthetised using 2.0% isoflurane. Tracheostomy was performed using a cannula. The bulla was opened using a retroauricular approach. An Ag–AgCl reference electrode was placed on the neck muscles, and a glass microelectrode filled with 2 M KCl was inserted into the scala media of the second turn to measure EP. The microelectrode was connected to an amplifier with high-input impedance (900 A Micropressure System; World Precision Instruments). All experiments were performed in an electrically shielded booth.

### Statistical analysis

All data are presented as the mean ± standard error. Statistical analysis of two groups was performed using two-tailed unpaired Student *t* test (Figs 2d,e and 3g,h, Table S1). When the variance between samples was unequal, Welch’s test was used (Fig. 2b). For comparisons of more than two groups, one-way analysis of variance followed by Tukey’s post-hoc test was performed (Figs 2c and 6d). A P value of <0.05 was considered statistically significant. All analyses were performed using StatMate Ver. 4.01 software (ATMS, Tokyo).

## Additional Information

**How to cite this article**: Kamitani, T. *et al.* Deletion of Tricellulin Causes Progressive Hearing Loss Associated with Degeneration of Cochlear Hair Cells. *Sci. Rep.* **5**, 18402; doi: 10.1038/srep18402 (2015).

## Supplementary Material

Supplementary Information
