# Supplementary material for: Deletion of Tricellulin Causes Progressive Hearing Loss Associated with Degeneration of Cochlear Hair Cells
Source: Sci Rep. 2015 Dec 18;5:18402. doi: 10.1038/srep18402 (PMC4683410; doi:10.1038/srep18402)
Supplement: Supplementary Information [file srep18402-s1.pdf]

Supplementary information

Deletion of Tricellulin Causes Progressive Hearing Loss Associated with Degeneration of Cochlear Hair Cells

Toru Kamitani[1, 2], Hirofumi Sakaguchi[2*], Atsushi Tamura[1], Takenori Miyashita[3], Yuji Yamazaki[1], Reitaro Tokumasu[1], Ryuhei Inamoto[3], Ai Matsubara[3], Nozomu Mori[3], Yasuo Hisa[2], Sachiko Tsukita[1]

*Correspondence and requests for materials should be addressed to H.S. (hiro-s@koto.kpu-m.ac.jp).

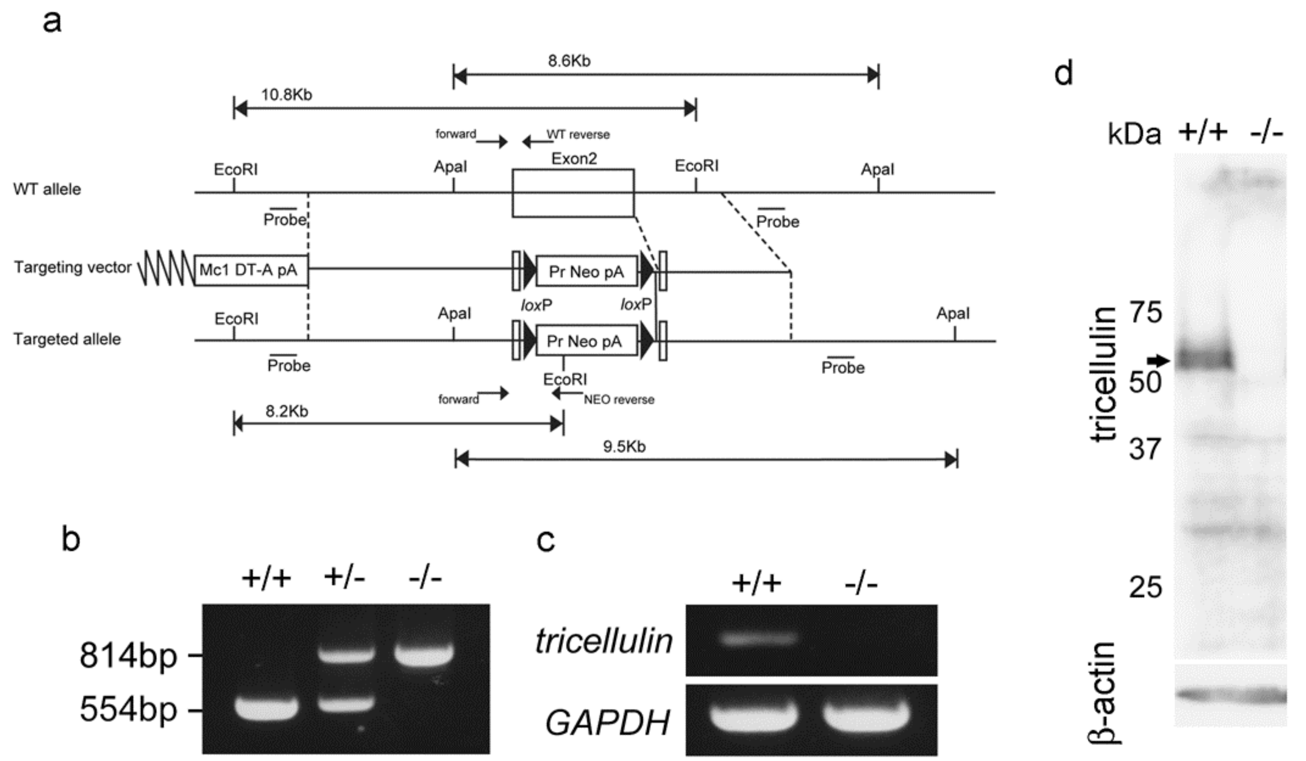

Figure S1. Generation of *Tric*$^{-/-}$ mice

(a) Diagrams of wild-type *Tric*, the targeting vector and the disrupted gene in the targeted allele. A diphtheria-toxin expression cassette (Mc1/DT-A) was linked to the 5′ end of the construct for negative selection. The locations of 5′ and 3′ probes for Southern blotting analysis and primers for PCR genotyping are indicated.

(b) PCR detection of *Tric* in *Tric*$^{+/+}$, *Tric*$^{+/-}$ and *Tric*$^{-/-}$ mice.

(c) RT-PCR showing the absence of *Tric* mRNA expression in *Tric*$^{-/-}$ mice.

(d) Western blotting analysis showing lack of detection of tricellulin expression (64 kDa: arrow) in *Tric*$^{-/-}$ mice. β-actin was used as the loading control.

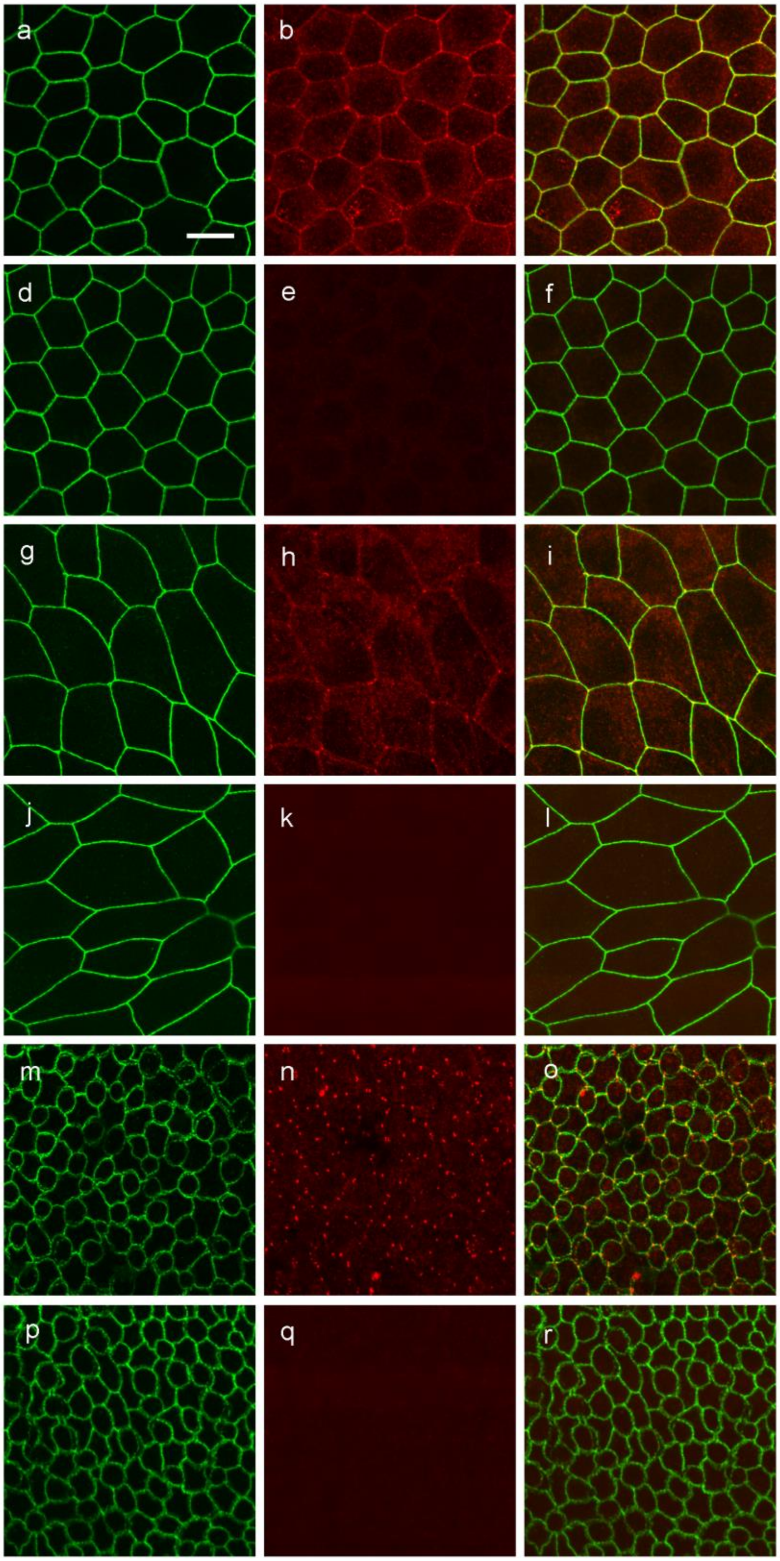
a
b
d
e
f
g
h
i
j
k
l
m
n
o
p
q
r

Figure S2. Tricellulin localisation in the stria vascularis, Reissner's membrane and utricle

(a–f) Marginal cells of the stria vascularis of *Tric*$^{+/+}$ (a–c) and *Tric*$^{-/-}$ mice (d–f) on P8.

(g–l) Epithelial cells of Reissner's membrane of *Tric*$^{+/+}$ (g–i) and *Tric*$^{-/-}$ mice (j–l) on P8.

(m-r) Hair cells of utricle of *Tric*$^{+/+}$ (m-o) and *Tric*$^{-/-}$ (p-r) on P8.

Tissues were immunostained with antibodies against ZO-1 (a–f, green), occludin (g–r, green) or tricellulin (a–r, red). Note that tricellulin localised to bTJs and tTJs of *Tric*$^{+/+}$ but not of *Tric*$^{-/-}$ mice.

Scale bar : 10 μm.

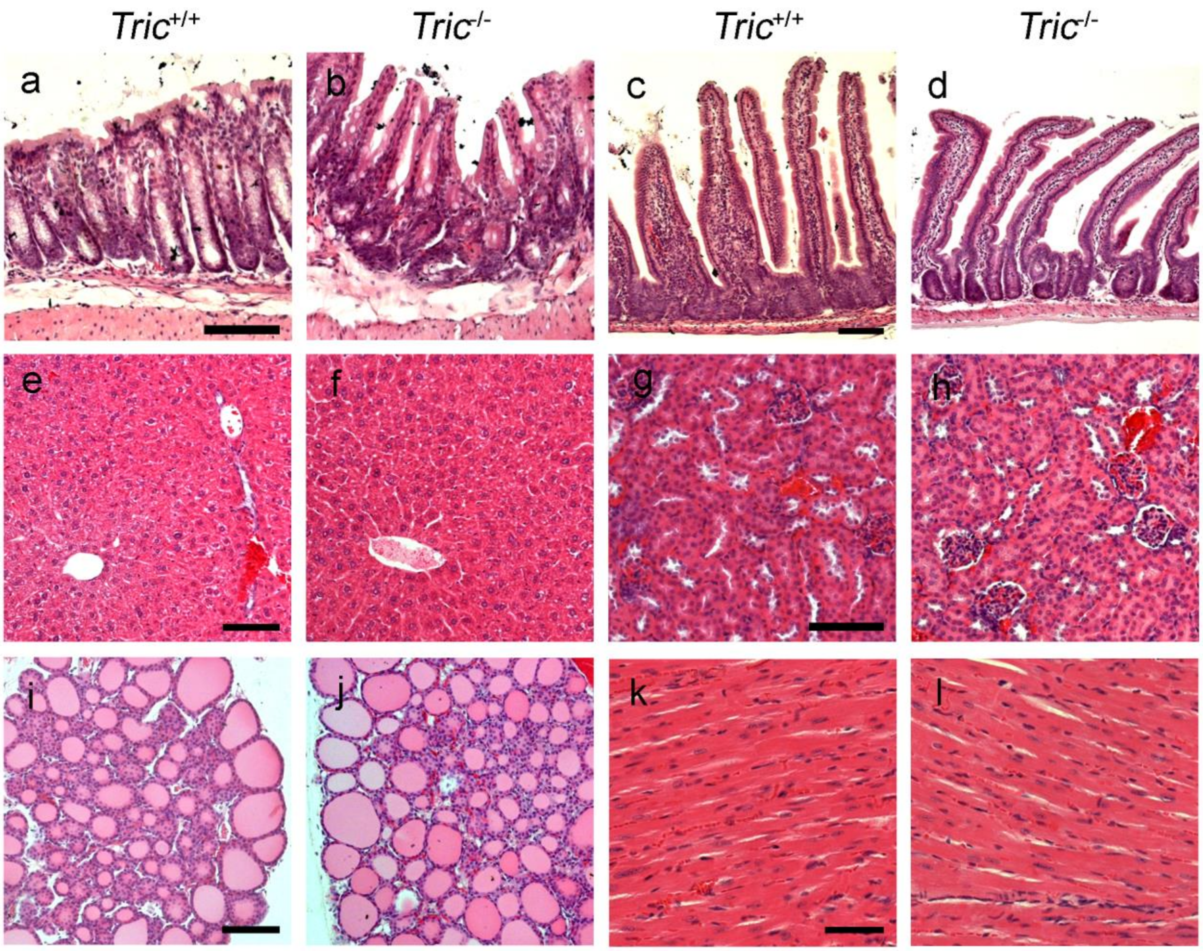

Figure S3. Absence of pathological phenotype in various tissues of *Tric*$^{-/-}$ mice

HE staining of colon (a, b), small intestine (c, d), liver (e, f), kidney (g, h), thyroid gland (i, j) and heart (k, l). No apparent difference is observed between *Tric*$^{+/+}$ (a, c, e, g, i, k) and *Tric*$^{-/-}$ (b, d, f, h, j, l) mice. Scale bars: 100 μm (a–j), 50 μm (k, l).

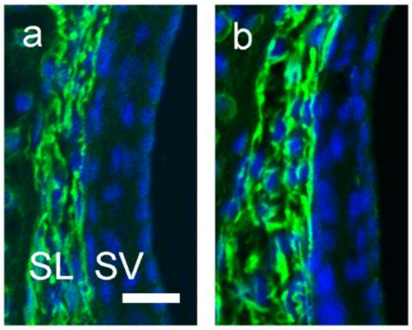

Figure S4. Paracellular impermeability of the stria vascularis is maintained in *Tric*$^{-/-}$ cochlea

(a) Biotin (green) perfused into the perilymph is distributed to the spiral ligament (SL) but not to the stria vascularis (SV) in *Tric*$^{+/+}$ cochlea. Nuclei were counterstained with DAPI (blue).

(b) Biotin is excluded from the stria vascularis of *Tric*$^{-/-}$ cochlea.

Scale bar = 20 μm.

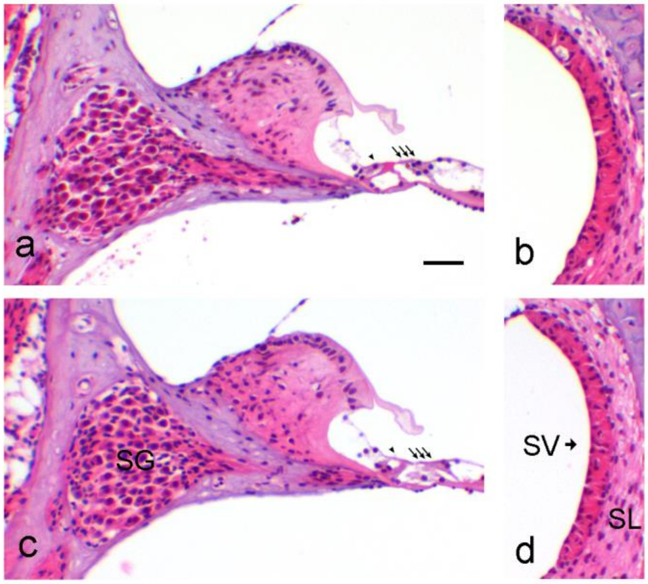

Figure S5. Loss of HCs from *Tric*$^{-/-}$ cochlea

(a) The organ of Corti of *Tric*$^{+/+}$ mice on P21 has one row of IHCs (arrowhead) and three rows of OHCs

(arrows).

(b) The stria vascularis and spiral ligament of *Tric*$^{+/+}$ mice.

(c) IHCs (arrowhead) and OHCs (arrows) are frequently lost, but the spiral ganglion (SG) is intact in *Tric*$^{-/-}$ mice.

(d) Normal morphology of the stria vascularis (SV) and spiral ligament (SL) of *Tric*$^{-/-}$ cochlea.

Scale bar = 100 μm.

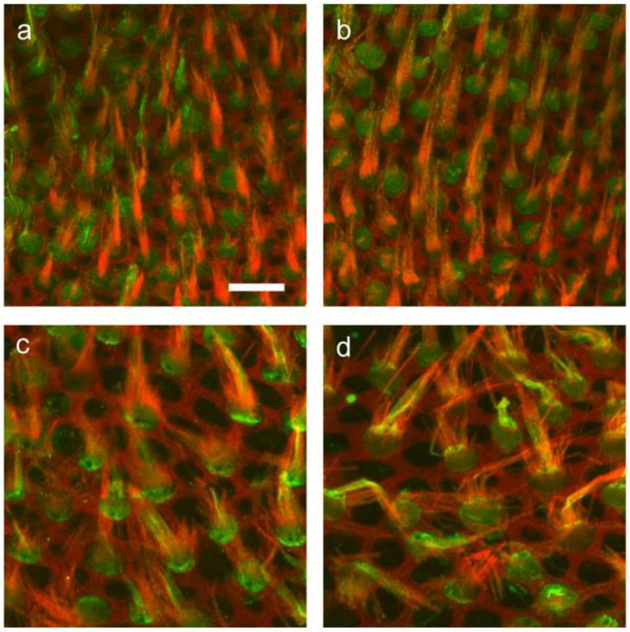

Figure S6. Absence of HC degeneration of the vestibular organs of *Tric*$^{-/-}$ mice

Utricles (a, b) and crista ampullaris (c, d) of *Tric*$^{+/+}$ (a, c) and *Tric*$^{-/-}$ mice (b, d) on P21 were labelled with an antibody against myosin VIIa (green) and with phalloidin (red). Scale bar = 10 μm.

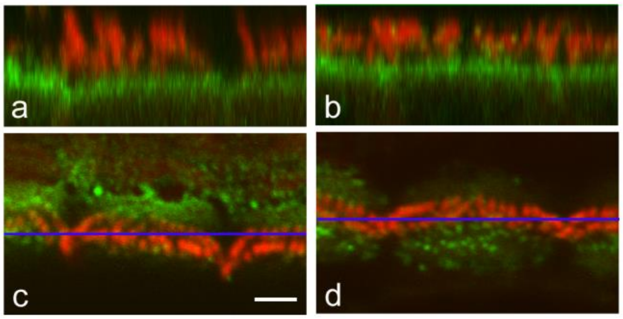

Figure S7. Normal apicobasal polarity in *Tric*$^{-/-}$ HCs

Vertical (a, b) and horizontal (c, d) views of IHCs labelled with an antibody against PKCζ (green) and with phalloidin (red). PKCζ accumulates at the apical surface of IHCs of *Tric*$^{+/+}$ (a, c) and *Tric*$^{-/-}$ (b, d) cochleae. The vertical views were obtained using image processing at blue lines. Scale bar = 2 μm.

Serum Chemistry	Units	*Tric*+/+	*Tric* −/−	P value
TP	g/dL	4.5±0.04	4.7±0.01	0.172
ALB	g/dL	3.1±0.08	3.4±0.1	0.092
BUN	mg/dL	37.3±2.8	38±5.6	0.921
CRE	mg/dL	0.075±0.01	0.073±0.02	0.936
Na	mEq/L	152.8±1.3	158.7±0.1	0.142
K	mEq/L	4.2±0.3	4.7±0.1	0.181
Cl	mEq/L	100±1.3	97.3±0.2	0.172
Mg	mg/dL	2.9±0.1	2.8±0.2	0.621
AST	IU/L	83.5±9.5	76.3±21.9	0.785
ALT	IU/L	36.3±6.9	36.3±12.0	0.996
ALP	IU/L	631±19.8	619.3±63.7	0.875
LDH	IU/L	323.8±103.2	227±77.3	0.487
AMY	IU/L	1632±64.6	1589.3±117.7	0.77
T-CHO	mg/dL	73±5.9	79±8.5	0.596
TG	mg/dL	66.3±20.6	31.7±8.4	0.197
LDL-C	mg/dL	10±1.4	13±2.7	0.388
HDL-C	mg/dL	32.8±3.3	39.7±5.8	0.369
T-BIL	mg/dL	0.07±0.01	0.06±0.01	0.922
GLU	mg/dL	246.3±13.6	208±22.3	0.228

Table S1. Biochemical analysis of sera of *Tric*$^{+/+}$ and *Tric*$^{-/-}$ mice

Data were obtained from *Tric*$^{+/+}$ and *Tric*$^{-/-}$ mice on P30. n = 4 for *Tric*$^{+/+}$, n = 3 for *Tric*$^{-/-}$. TP : total protein, ALB : albumin, BUN : blood urea nitrogen, CRE : creatinine, Na : sodium, K : potassium, Cl : Chloride, Mg : magnesium, AST : asparatate aminotransferase, ALT : alanine aminotransferase, ALP : alkaline phosphatase, LDH : lactate dehydrogenase, AMY : amylase, T-CHO : total cholesterol, TG : triglyceride, LDL-C : low density lipoprotein cholesterol, HDL-C : high density lipoprotein cholesterol, T-BIL : total bilirubin, GLU : glucose.
